# PHL3/364: Patient Information via Faxback, Web & Compact Disc

**DOI:** 10.2196/jmir.1.suppl1.e84

**Published:** 1999-09-19

**Authors:** C Adams

**Affiliations:** ^1^North East Valley Division of General Practice, VictoriaAustralia

**Keywords:** Medical Informatics, Web, Compact Disc, Patient Information

## Abstract

**Introduction:**

As part of our information technology initiative our division has collated a wide range of patient information sheets in General Health and Travel. We have made these available to our members via "Faxback", Web site and Compact Disc. Instructive and informative videos are a recent addition.

**Methods:**

Quality information from organisations such as the Heart Foundation, the Arthritis Foundation, the Lung Foundation and the Gastroenterology Institute have been available for many years in pamphlets. Our initial project was to collect and distribute this material. This resource was rarely used. With the permission of these organisations, the division copied selected material into Word document format and placed this on a "Faxback" computer. Our members were provided with a laminated Faxback index sheet. Calls to "Faxback" were monitored. We have since placed all this information on our Web site and a Compact Disc (Released in May 1999). The reason we chose to use HTML format is that it allows for easy navigation of information sheet material, as well as the ability to use the Web browser off-line; make links to further information from within an information sheet; access links to other Web sites on-line; use other Web sites placed on CD (with permission); use a search engine off-line to find files; provide high quality graphics, animations and "talking pictures"; access videos for procedural instruction and patient information. We also included a copy of our very popular Faxback information index on the CD which links to the relevant Word 7 document. These Word 7 document format sheets are more compact than html and most can be printed out on a single A4 sheet of paper (i.e., back to back).

**Results:**

The above can be demonstrated using a computer projector.

**Discussion:**

General Practitioners have limited time to research information for themselves and their patients. The success of "Faxback" indicates a willingness of the conservative GP to accept information technology if it is easy to use, and provides what they want. Our Web site provides a large collection of quality information and has been acclaimed as easy to navigate. However the world wide web is considered too slow by most GP's, and is rarely used during office hours. The Compact Disc aims to educate and familiarise the General Practitioner with the benefits of web technology and provide a convenient and quick method of access to patient information for use in office hours.

